# Supplementary material for: A generalized epilepsy network derived from brain abnormalities and deep brain stimulation
Source: Nat Commun. 2025 Mar 24;16:2783. doi: 10.1038/s41467-025-57392-7 (PMC11933423; doi:10.1038/s41467-025-57392-7)
Supplement: Supplementary file 1 — Supplementary Information [file 41467_2025_57392_MOESM1_ESM.pdf]

Supplementary materials for

A generalized epilepsy network derived from brain abnormalities and deep brain stimulation

Corresponding authors

Frederic L.W.V.J. Schaper MD PhD
60 Fenwood Road, Boston, MA 02115, U.S.
email: fredericschaper@icloud.com

and

Kai Wang MD PhD
No.81 Meishan Road, Shushan District, Hefei, 230032, China,
email: wangkai1964@126.com

Supplementary Methods

Supplementary Methods 1

Coordinates

We systematically searched the published literature for neuroimaging studies using voxel-based-morphometry to identify structural MRI changes and resting-state fMRI studies to identify functional MRI changes in IGE patients versus healthy controls.

In line with the best-practice recommendations for coordinate-based meta-analysis [1], two independent investigators performed a systematic literature search to identify coordinates of neuroimaging abnormalities associated with IGE. Any discrepancies in literature search, record evaluation or selection, and data extraction between the two investigators were resolved by concensus. The following search terms were used in PubMed and EMBASE databases: "voxel-based morphometry", "resting state functional MRI", and "epilepsy". A detailed description of the search strategy can be found in **Supplementary Table 1**. Inclusion criteria were: (a) English language, published and peer-reviewed studies, (b) published coordinates of structural or functional neuroimaging abnormalities, (c) patients were diagnosed with IGE according to ILAE criteria, (d) a comparison between patients with epilepsy and healthy controls was made, (e) a voxel-based whole-brain analysis was performed, (f) using VBM in the structural MRI studies and measures of local activity (such as ALFF [2] and ReHo [3]) in the functional MRI studies. The exclusion criteria were: (a) voxel-based study was only performed within a small region of interest, (b) fMRI study did not compute measures in the conventional low frequency (0.01 to 0.1 Hz), or (c) study did not report any significant coordinates which could also not be retrieved after contacting the corresponding authors. Finally, we included a sample of 21 studies from 20 papers **(Supplementary Figure 1)**. Here, the "study" refers to a single independent analysis or contrast in a given paper with different control groups. For each study, the reported Montreal Neurological Institute (MNI) coordinates of the significant regions of atrophy or fMRI hyperactivity were retrieved. Coordinates reported in Talairach space were converted into MNI space using GingerALE software (version 3.0.2).

Supplementary Methods 2

Normative connectome

Functional connectivity data were obtained at the University of Science and Technology of China (Hefei, China) with a 3-T scanner (Discovery 750; GE Healthcare, Milwaukee, WI, USA). 652 participants (316 males and 336 females) were instructed to rest with their eyes closed without falling asleep.

High resolution T1-weighted images were acquired in the sagittal orientation using a three-dimensional brain-volume sequence (repetition/echo time, 8.16/3.18 ms; flip angle, 12; field of view, 256 × 256 mm^2; 256 × 256 matrix; section thickness, 1 mm; voxel size, 1 × 1 × 1 mm^3). Resting-state functional images were acquired using a single shot gradient-recalled echo planar imaging sequence (repetition/echo time, 2400/30 ms; flip angle, 90; field of view, 192 × 192 mm^2; 64 × 64 in-plane matrix; section thickness, 3 mm; voxel size, 3 × 3 × 3 mm^3; 46 transverse sections). A total of 217 volumes were acquired (~8.7 mins).

The rs-fMRI data were preprocessed using WhiteMatter software (https://github.com/jigongjun/Neuroimaging-and-Neuromodulation) that combined functions in SPM12 software (www.fil.ion.ucl.ac.uk/spm) and ANFI (https://afni.nimh.nih.gov/afni/). Noise variables from motion, CSF, white matter, and the global signal were regressed out in the preprocessing. Specifically, the preprocessing steps were as follows: 1) delete the first five time points; 2) remove temporal spikes; 3) slice timing and head motion correction; 4) co-registration to the structural image; 5) regress out nuisance regressors (24 head motion parameters, and average signals in the cerebrospinal fluid, white matter, and whole brain); 6) spatial normalization to the MNI space using the matrix produced by structural image segmentation (DARTEL algorithm) [38]; and 7) spatial smoothing with a 4-mm full width at half-maximum Gaussian kernel.

Supplementary Methods 3

IGE connectome

Functional connectivity data of 172 patients with IGE were collected in two sites, Hefei and Nanjing, China.

MRI data of 52 patients (31 males and 21 females; age, 30.0±10.74 years) were obtained at the First Affiliated Hospital of Anhui Medical University (Hefei, China) with Siemens 3-T MRI Scanner Prisma. Participants were instructed to rest with their eyes closed without falling asleep. High resolution T1-weighted images were acquired in the sagittal orientation using a magnetization-prepared rapid gradient-echo sequence (repetition/echo time, 2300/2.96 ms; flip angle, 9; field of view, 240 × 256 mm^2; 240 × 256 matrix; section thickness, 1 mm; voxel size, 1 × 1 × 1 mm^3). Resting-state functional images were acquired using a single shot gradient-recalled echo planar imaging sequence (repetition/echo time, 3000/30 ms; flip angle, 90; field of view, 220 × 220 mm^2; 64 × 64 in-plane matrix; section thickness, 3.4 mm; voxel size, 3.4 × 3.4 × 3.4 mm^3; 48 transverse sections). A total of 217 volumes were acquired (~8.7 mins).

The other data of 120 patients (81 males and 39 females; age, 25.5±8.68 years)

were acquired on a clinical 3-T MR scanner (TIM Trio; Siemens Medical Solutions, Erlangen, Germany) at Jinling Hospital (NanJing, China). Functional images were acquired by using a single shot, gradient-recalled echo-planar imaging sequence (repetition time msec/echo time msec, 2000/30; flip angle, 90°, voxel size, 3.75 × 3.75 × 4.4 mm^3, 250 volume), aligned along the anterior–posterior commissure line were acquired for each subject, a total of 250 volumes were acquired, The high-spatial-resolution three-dimensional T1-weighted anatomic images were acquired in sagittal orientation by using a magnetization-prepared rapid gradient-echo sequence (repetition/echo time, 2300/2.98; flip angle, 9°; voxel size, 1 × 1 × 1 mm^3; sections, 176). See details of the diagnosis and scanning parameters in our previous work [4-6].

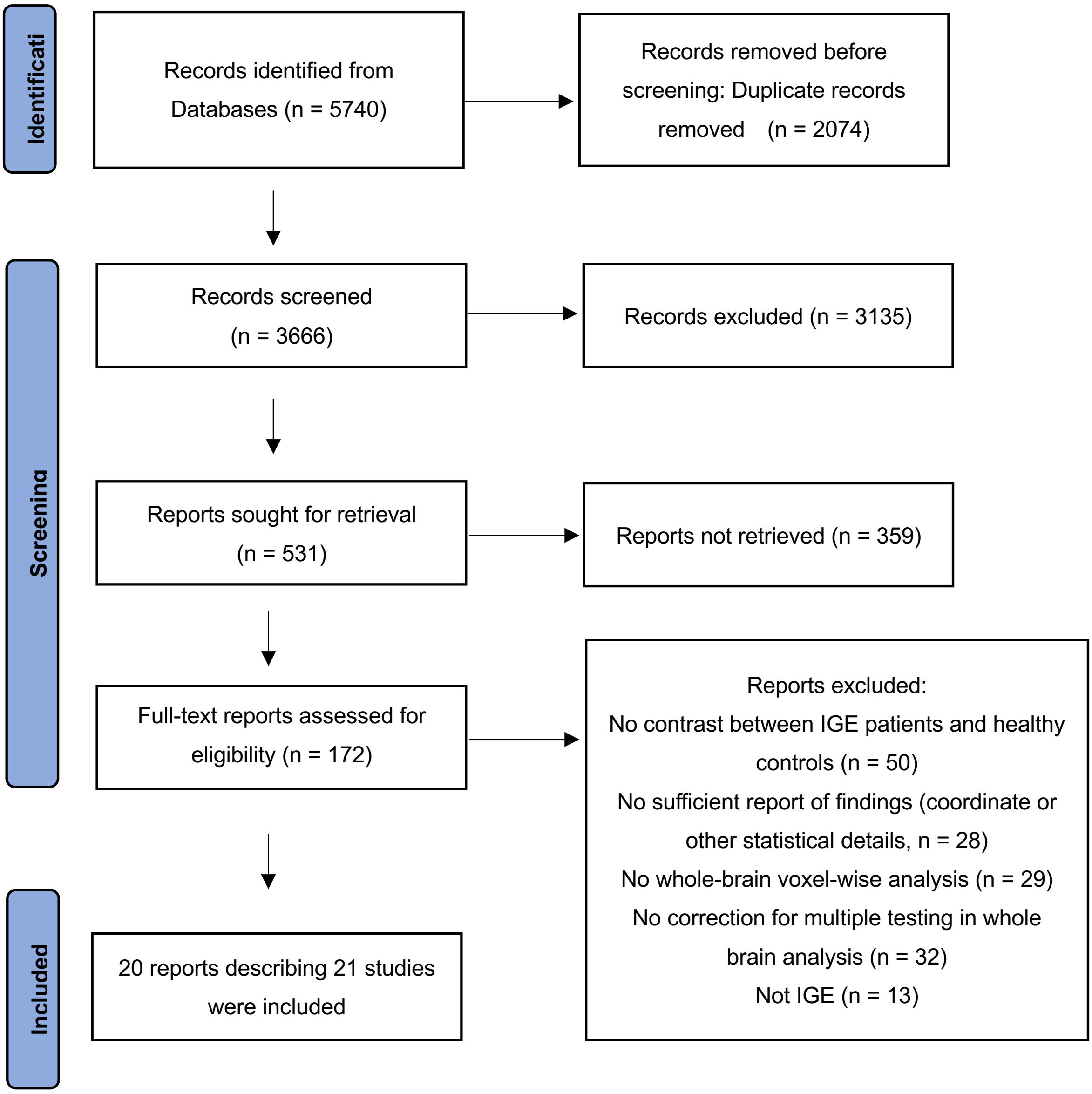

Supplementary Figure 1. Flow diagram of systematic literature search. IGE = Idiopathic generalized epilepsy.

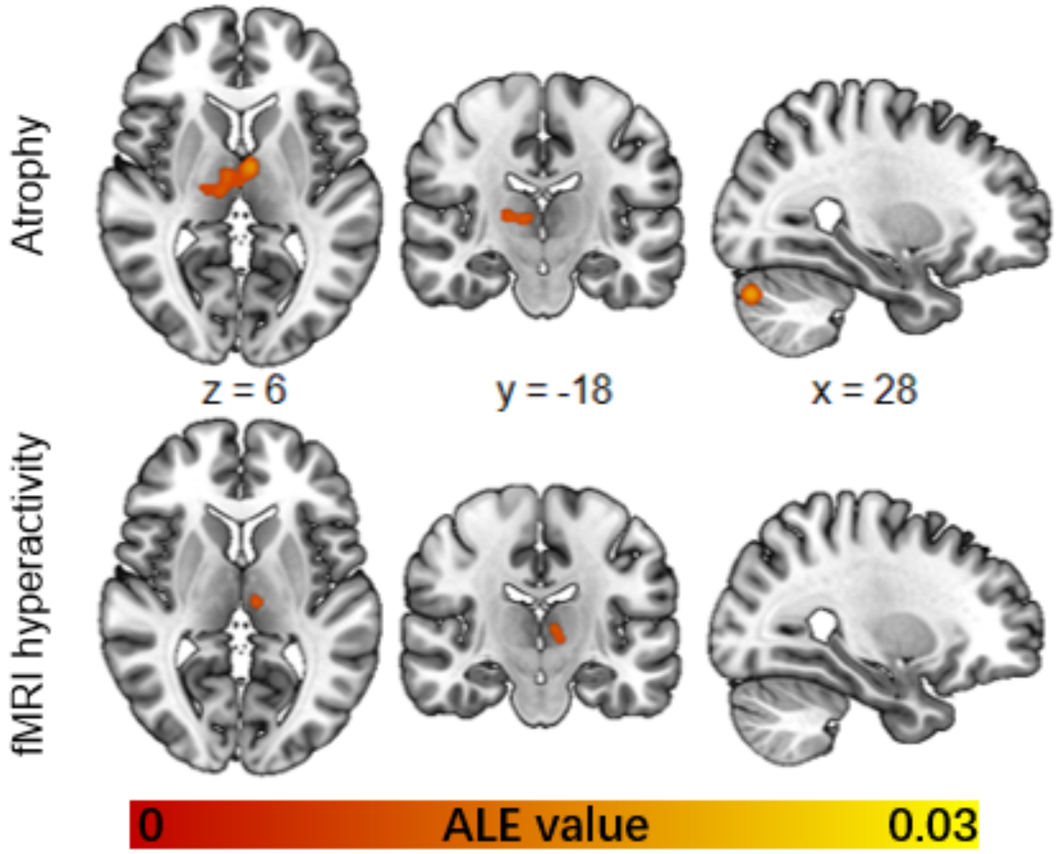

Supplementary Figure 2. ALE meta-analysis performed separately for coordinates of brain atrophy or fMRI hyperactivity identified different locations in the thalamus and cerebellum.

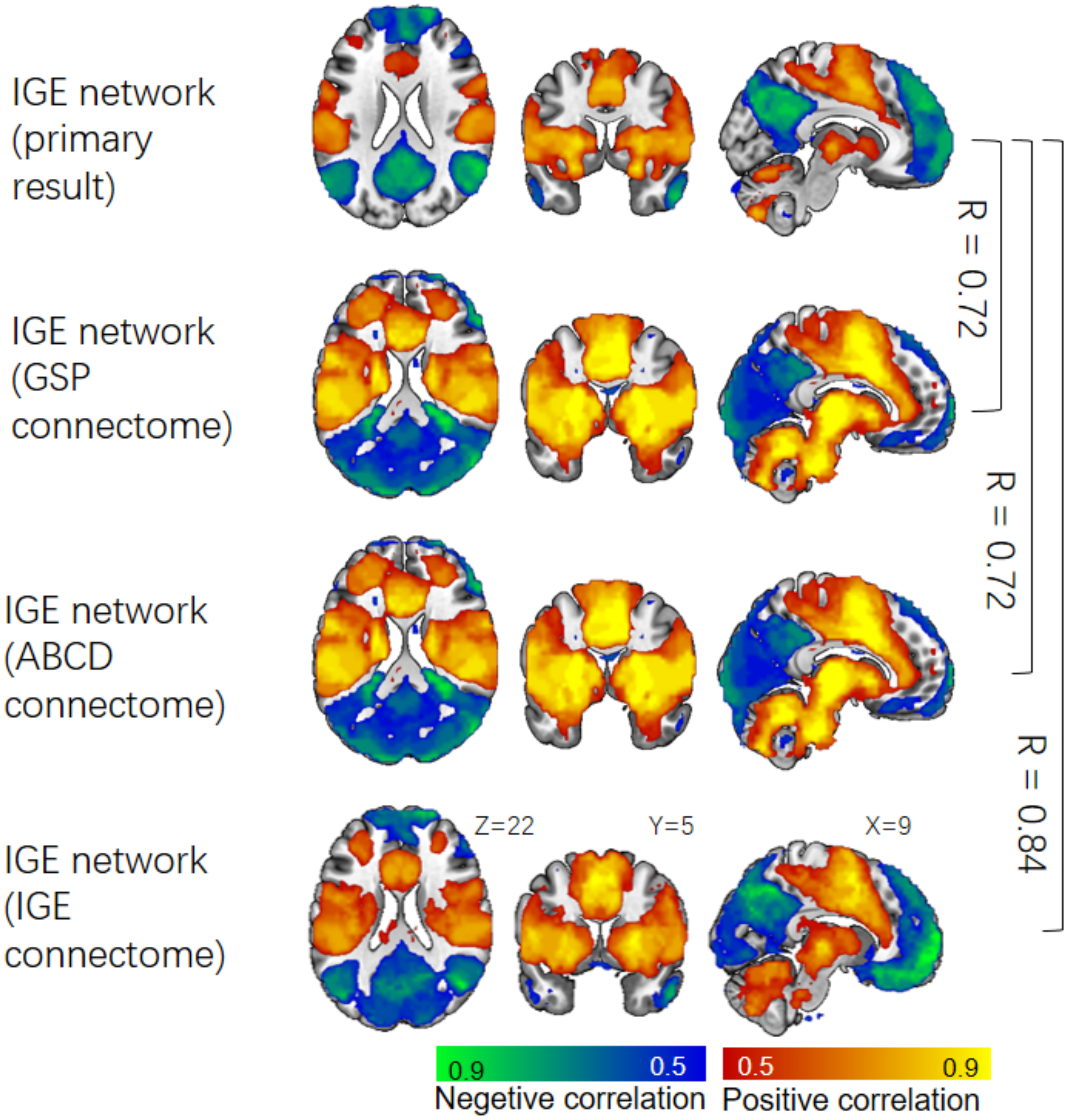

Supplementary Figure 3. An independent normative adult connectome (GSP), pediatric connectome (ABCD), and disease-specific IGE connectome identified a similar IGE network (average spatial r = 0.76). IGE networks derived from both the adult and pediatric connectome were based on *t* threshold of 9 due to the large sample size (n = 1000 in both connectomes). The *t* threshold used for the IGE connectome was 2 due to a smaller sample size in this disease-specific connectome (n = 172 patients with IGE).

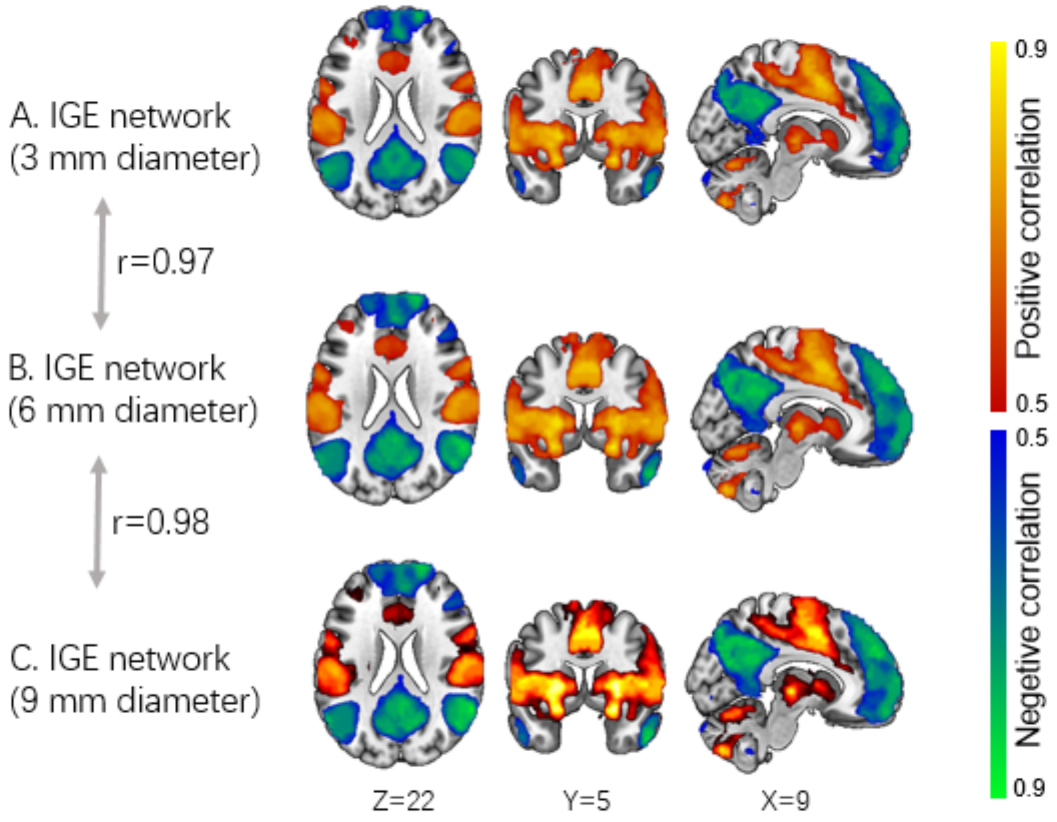

Supplementary Figure 4. Coordinate network mapping analysis using different sphere sizes identified a similar IGE network (average spatial r = 0.975).

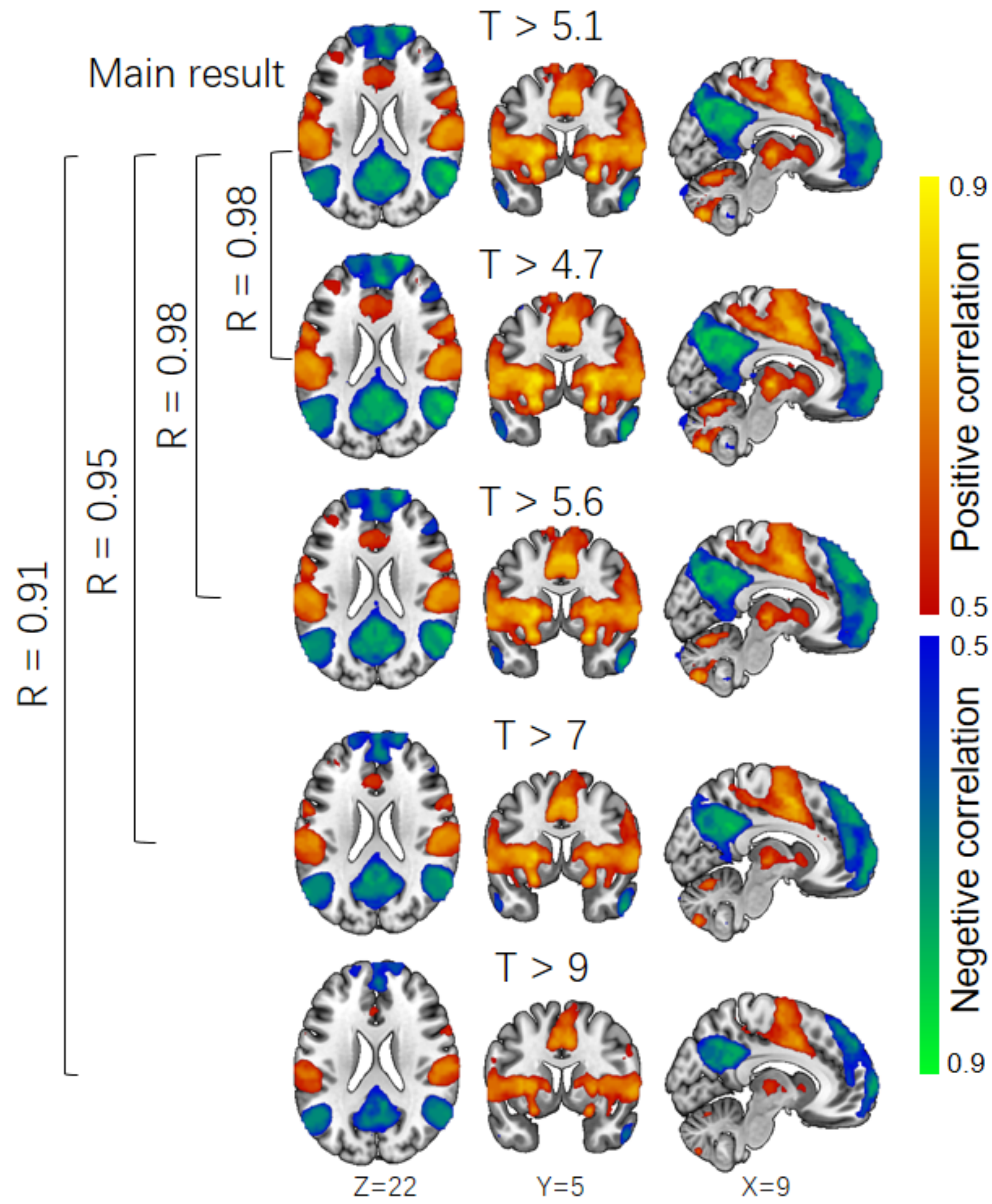

Supplementary Figure 5. Coordinate network mapping analysis at different *t* thresholds identified a similar IGE network suggesting our network results are independent on arbitrary statistical thresholds (average spatial r = 0.96).

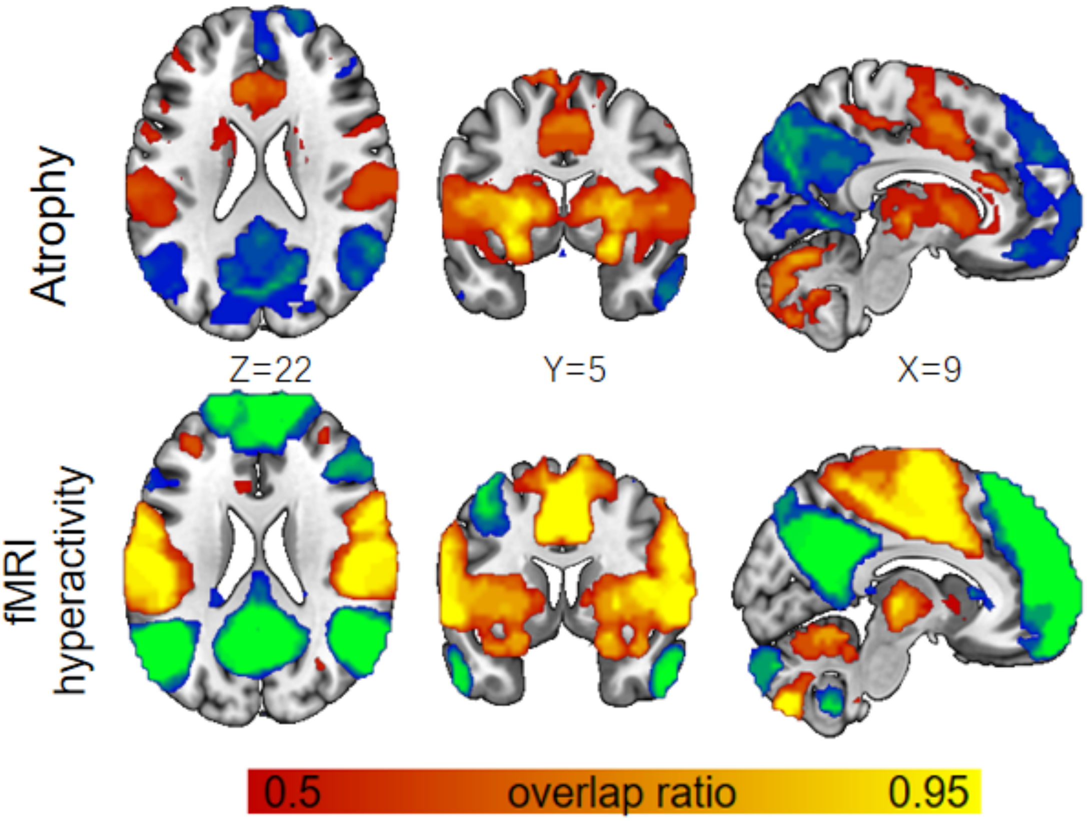

Supplementary Figure 6. Coordinate network mapping analysis performed separately for study level coordinates of brain atrophy (n=13) and fMRI hyperactivity (n=8) identified a similar IGE network (Spatial r = 0.63).

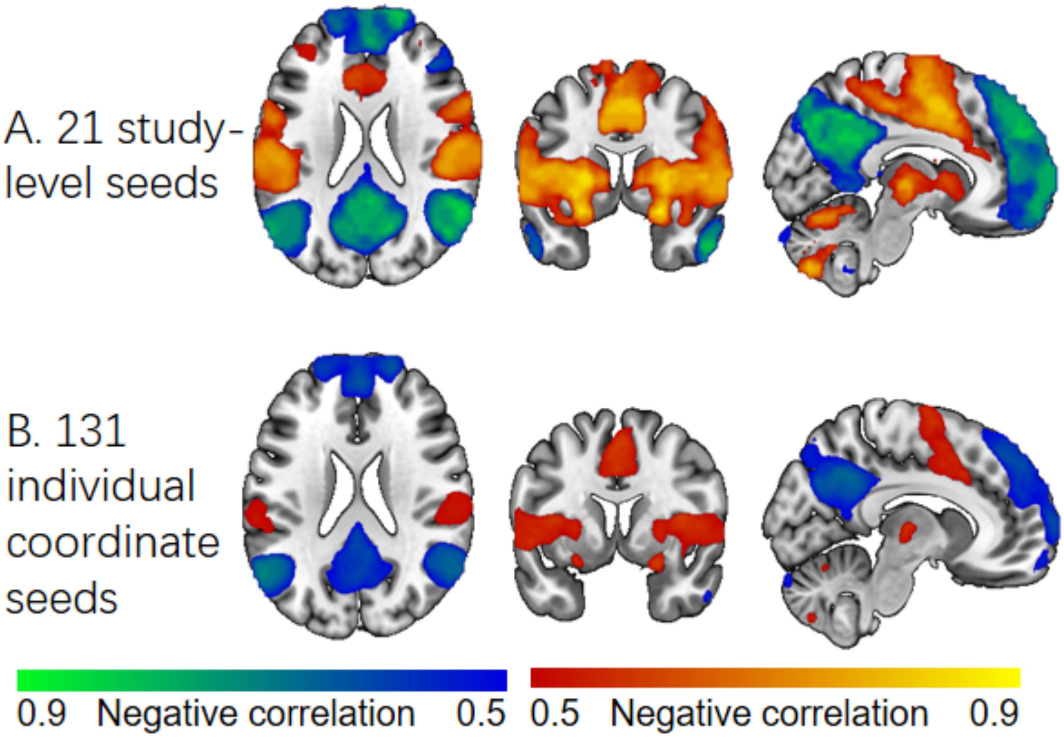

Supplementary Figure 7. The IGE network recreated using each individual coordinate (n=131) as a seed was highly similar (spatial r = 0.91) to the IGE network generated from study-level seeds (n=21).

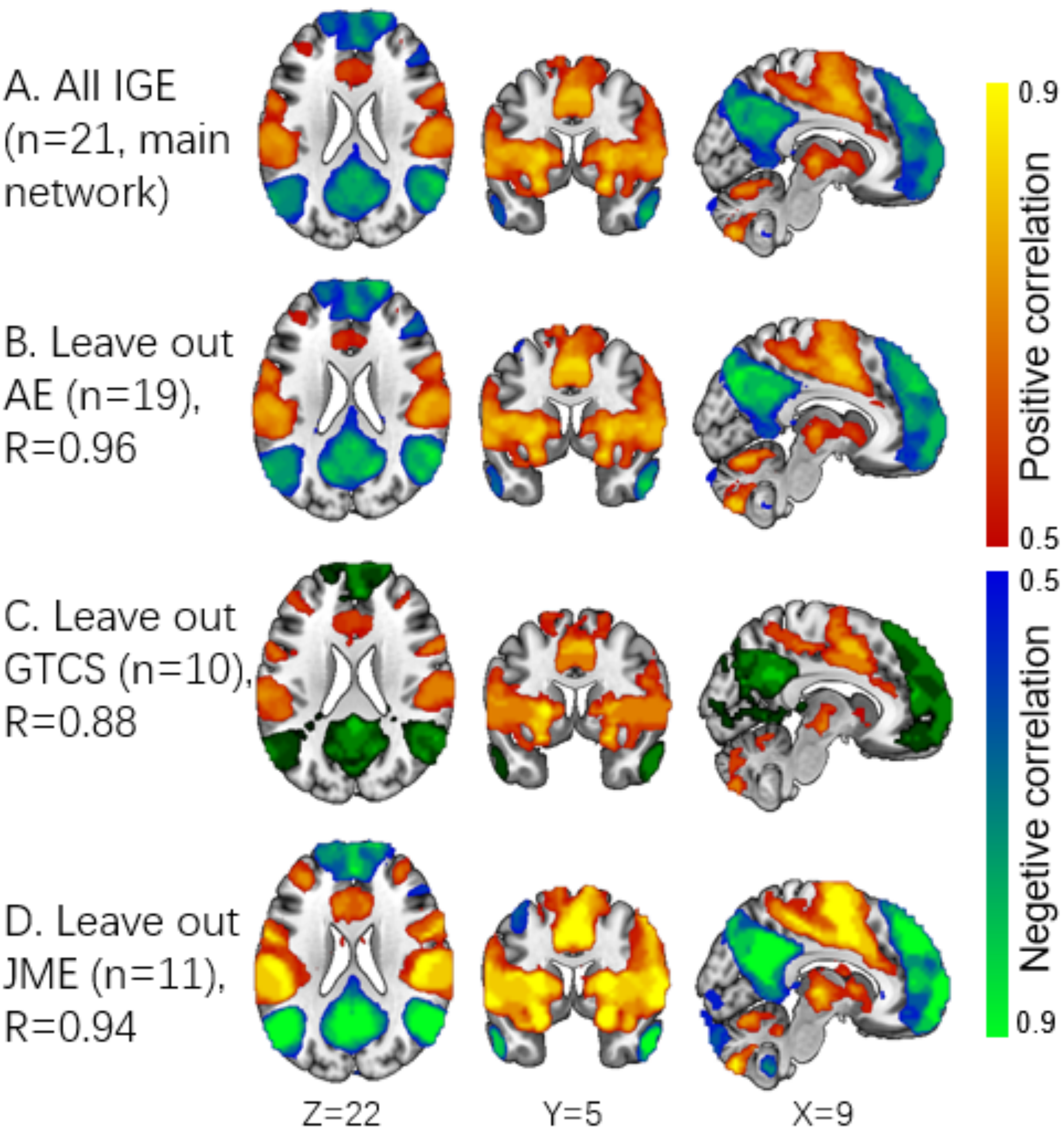

Supplementary Figure 8. The IGE network (A) was not driven by any particular IGE 'sub'-syndrome in a leave-one-diagnosis out analysis (B-D, average spatial R = 0.93). *Abbreviations: AE; absence epilepsy, GTCS; generalized tonic clonic seizures, JME; juvenile myoclonic epilepsy.*

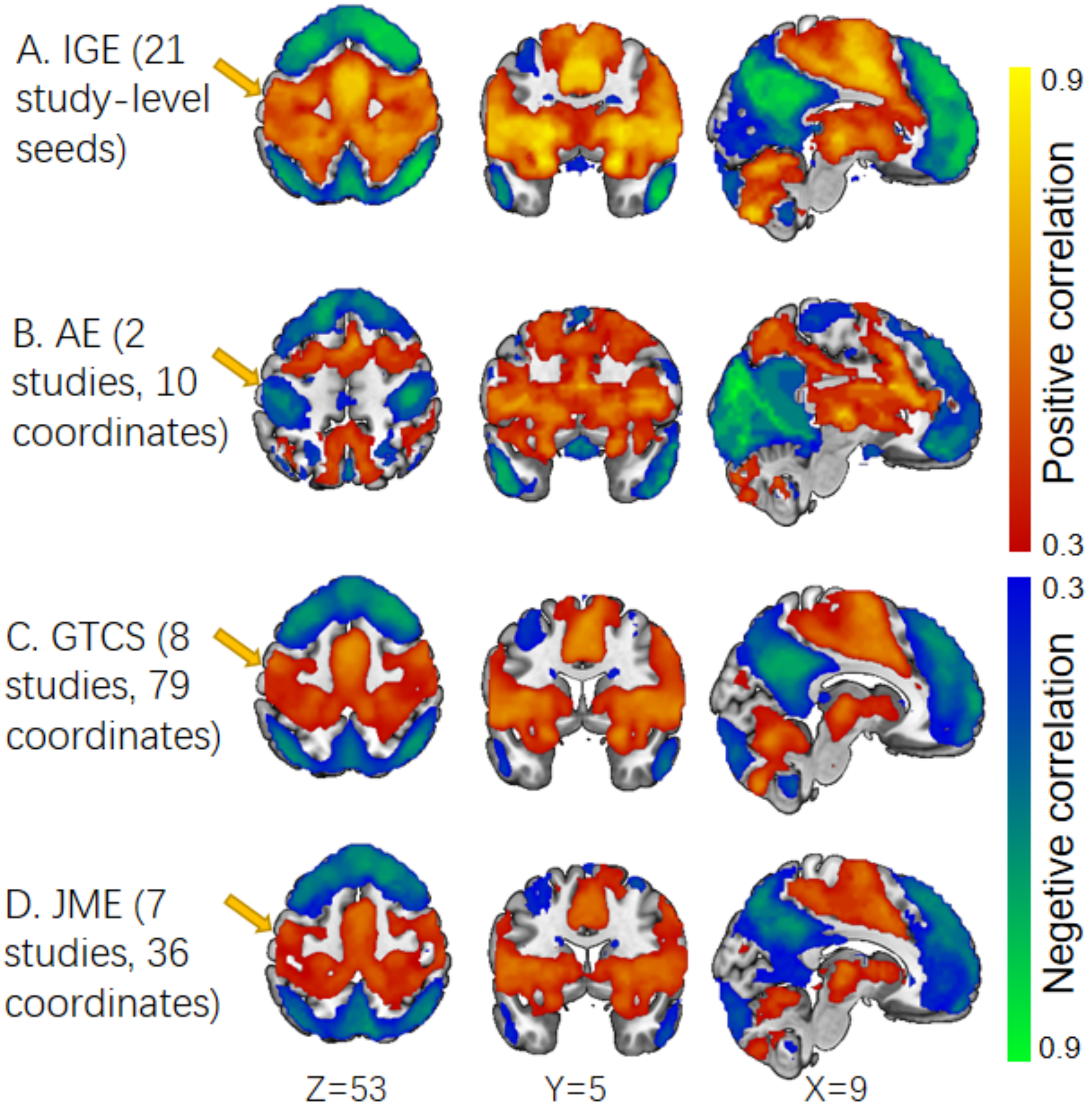

Supplementary Figure 9. In a subgroup analysis, the coordinate network mapping analysis was repeated for each IGE sub-syndrome. To increase power with the inherent reduced sample size in subgroup analyses, each individual coordinate was used as a seed instead of the study level coordinates. The subcortical connectivity profile was similar between subgroups, but the cortical connectivity profile differed slightly. AE coordinates showed negative functional connectivity to the motor cortex (yellow arrow), while JME and GTCS coordinates showed positive functional connectivity (albeit not statistically significant in a voxel-wise two sample *t*-test). Note that 4 studies included multiple different IGE sub-syndromes and were therefore excluded from this analysis (see Supplementary Table 2 and 3). *Abbreviations: AE; absence epilepsy, GTCS; generalized tonic clonic seizures, JME; juvenile myoclonic epilepsy.*

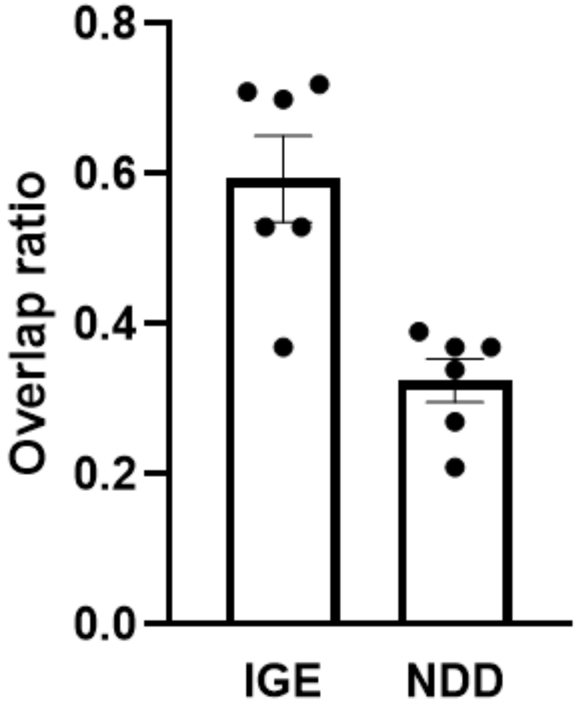

Supplementary Figure 10. Coordinates of hyperactivity in EEG-fMRI studies of generalized epilepsies overlapped more with our IGE network than a coordinate network map for neurodegenerative disease (NDD) (paired t = 8.34, P = 0.0004). Data are presented as mean values +/- SEM.

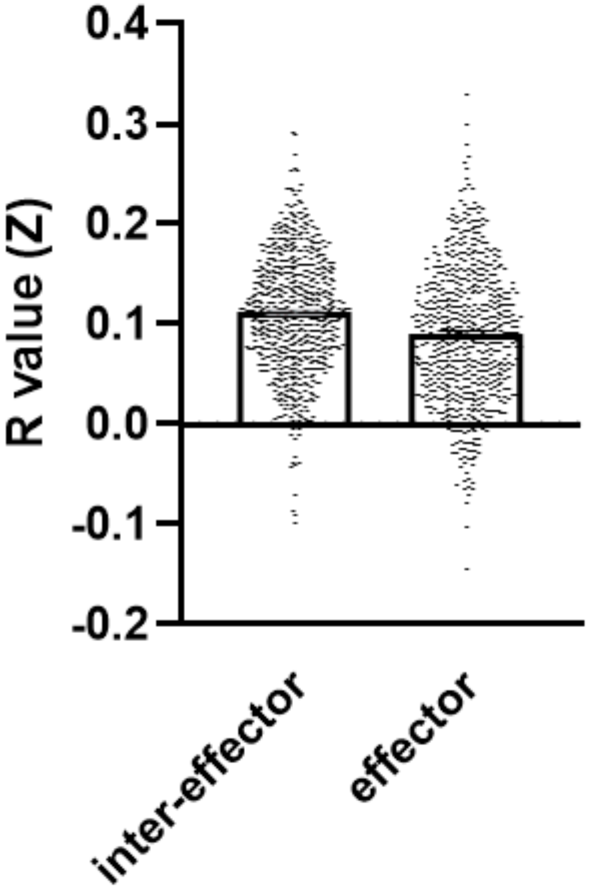

Supplementary Figure 11. Study-level IGE coordinates show higher functional connectivity to the inter-effector regions than the effector regions of the M1 homunculus (paired t = 8.84, P < 0.0001). Data are presented as mean values +/- SEM.

Supplementary Table 1. Search strategies for structural and functional studies of IGE.

Structural MRI	
Database	PubMed
Data of search	Initial search November 2019, updated January 2020
Search strategy	Search item:<br>(Epilepsy[Title/Abstract] AND(((((voxel[Title/Abstract])OR(voxelwise[Title/Abstract])) OR(voxel-based[Title/Abstract]))OR(VBM[Title/Abstract]))OR(morphometry[Title/Abstract]))) Filters: from 1977-2020 Sort by: First Author<br>Publication data :From 1977.1.1<br>Species:Humans<br>Language:English
Items found	615
Database	Embase
Data of search	Initial search November 2019, updated January 2020
Search strategy	Search item #1: 'Epilepsy':ab,ti<br>Search item #2: 'voxel':ab,ti OR 'voxelwise':ab,ti OR 'VBM':ab,ti OR 'voxel-based':ab,ti OR 'voxelwise':ab,ti OR 'morphometry':ab,ti<br>Search item #3: #1 AND #2<br>Publication data: From 1977.1.1<br>Species:Humans<br>Language:English
Items found	1017
**Functional MRI**	
Database	PubMed
Data of search	Initial search November 2019, updated January 2020
Search strategy	Search item:<br>(Epilepsy[Title/Abstract] AND(((((((fmri[Title/Abstract]) OR (rs-fmri[Title/Abstract])) OR (resting-state[Title/Abstract]))

	OR (alff[Title/Abstract])) OR (reho[Title/Abstract])) OR (functional magnetic resonance imaging[Title/Abstract])) OR (rest-state fmri[Title/Abstract]))) Filters: from 1976-2020<br>Sort by: First Author<br>Publication data :From 1976.1.1<br>Species:Humans<br>Language:English
Items found	1566
Database	Embase
Data of search	Initial search November 2019, updated January 2020
Search strategy	Search item #1: 'Epilepsy':ab,ti<br>Search item #2: 'fmri':ab,ti OR 'rs-fmri':ab,ti OR 'resting-state':ab,ti OR 'alff':ab,ti OR 'reho':ab,ti OR 'functional magnetic resonance imaging':ab,ti OR 'rest-state fmri':ab,ti<br>Search item #3: #1 AND #2<br>Species:Humans<br>Language:English
Items found	2542

Supplementary Table 2. Detailed information for structural MRI studies of IGE.

Reference	Sample size	Seizure type	IGE syndrome	Measure	P value
Chan et al，2006 [7]	N_p=10, N_{con}=109	AS	CAE	GMV	P_{corr}<0.05
Kim et al，2007 [8]	N_p=25, N_{con}=44	Myoclonic seizure and GTCS, with or without AS	JME	GMV	P_{corr}<0.05
de Araújo Filho et al，2009 [9]	N_p=38, N_{con}=30	Generalized spike and wave or poly-spike	JME	GMV	P_{corr}<0.05

		and wave activity			
Huang et al，2011 [10]	N_p=31, N_{con}=37	GTCS	GTCS	GMV	P_{corr}<0.05
Liu et al，2011 [11]	N_p=15, N_{con}=15	GTCS	JME	GMV	P_{corr}<0.05
Liu et al，2011 [11]	N_p=10, N_{con}=10	GTCS	GTCS	GMV	P_{corr}<0.05
O'Muircheartaigh et al，2011 [12]	N_p=28, N_{con}=55	Myoclonic jerks, GTCS	JME	GMV	P_{corr}<0.05
Kim et al，2013 [13]	N_p=50, N_{con}=50	AS, myoclonic seizure, GTCS	JME, GTCS, JAE	GMV	P_{corr}<0.05
Kim et al，2014 [14]	N_p=49, N_{con}=42	AS, myoclonic seizure, GTCS	JME, GTCS, JAE	GMV	P_{corr}<0.05
Lin et al，2009 [15]	N_p=60, N_{con}=30	Myoclonia, AS, GTCS	JME	GMV	P_{corr}<0.01
Wang et al，2018 [16]	N_p=14, N_{con}=30	GTCS	GTCS	GMV	P_{corr}<0.05
Zeng et al，2015 [17]	N_p=17, N_{con}=15	No seizure information	BAFME	GMV	P_{corr}<0.05
Zhong et al，2018 [18]	N_p=25, N_{con}=24	No seizure information	JME	GMV	P_{corr}<0.05

BAFME = Benign adult familial myoclonic epilepsy; CAE = Childhood absence epilepsy; FOCA = Four-dimensional consistency of local neural activities; GMV = Gray matter volume; GTCS = Generalized tonic-clonic seizures; IGE = Idiopathic generalized epilepsy; JAE = Juvenile absence epilepsy; JME = Juvenile myoclonic epilepsy; N_P = Number of patients; N_{con} = Number of controls.

Supplementary Table 3. Detailed information for resting-state functional MRI studies of IGE.

Reference	Sample size	Seizure type	IGE syndrome	Measure	P value
Zhong et al., 2011[19]	N_p=25, N_{con}=25	GTCS	GTCS	ReHo	P_{corr}<0.05
Jiang et al., 2016[20]	N_p=21, N_{con}=22	GSWD or polyspike-wave discharges	JME	ReHo	P_{corr}<0.05
Zhu et al., 2016[21]	N_p=70, N_{con}=70	GTCS	GTCS	FCD	P_{corr}<0.05
Ma et al., 2017[22]	N_p=19, N_{con}=22	GTCS	GTCS	FOCA	P_{corr}<0.05
Jia et al., 2018[23]	N_p=60, N_{con}=60	GTCS	JME, GTCS	ALFF	P_{corr}<0.05
Wang et al., 2018[16]	N_p=14, N_{con}=30	GTCS	GTCS	fALFF	P_{corr}<0.05
Liu et al., 2019[24]	N_p=28, N_{con}=28	GTCS	GTCS	ReHo	P_{corr}<0.05
Yan et al., 2020[25]	N_p=30, N_{con}=30	AS	CAE	fALFF	P_{corr}<0.05

AS = absence seizure; CAE = childhood absence epilepsy; FOCA = FOur-dimensional Consistency of local neural Activities; GTCS = generalized tonic-clonic seizures; IGE = Idiopathic Generalized Epilepsy; JME = juvenile myoclonic epilepsy; N_P = Number of patients; N_{con} = Number of controls.

Supplementary Table 4. Activation likelihood estimation clusters.

Brain regions	MNI coordinate	ALE value	Z value	P value
Thalamus, Medial Dorsal Nucleus	6, -12, 8	0.02	5.60	1.09E-08
Thalamus, Ventral Posterior Lateral Nucleus	20, -18, 6	0.02	4.62	1.91E-06
Thalamus	-4, -6, 6	0.02	4.56	2.59E-06
Thalamus	-10, -22, 2	0.02	4.09	2.12E-05
Thalamus, Medial Dorsal Nucleus	-6, -16. 8	0.01	3.85	5.98E-05

Supplementary Table 5. Control coordinate network studies associated with neurodegenerative disease.

	Reference	Imaging Modality	Contrast
**Alzheimer's Disease (AD)**			
1	Baron et al., 2001 [26]	MRI, atrophy	AD vs Controls
2	Boxer et al., 2003 [27]	MRI, atrophy	AD vs Controls
3	Boxer et al., 2003 [28]	MRI, atrophy	AD vs Controls
4	Bozzali et al., 2006 [29]	MRI, atrophy	AD vs Controls
5	Grossman et al., 2004 [30]	MRI, atrophy	AD vs Controls
6	Ohnishi et al., 2001 [31]	MRI, atrophy	AD vs Controls
7	Irish et al., 2013[32]	MRI, atrophy	AD vs Controls
8	Zahn et al., 2005 [33]	MRI, atrophy	AD vs Controls
**Frontotemporal Dementia (FTD)**			
1	Amanzio et al., 2016 [34]	MRI, atrophy	FTD vs Controls
2	Baez et al., 2016 [35]	MRI, atrophy	FTD vs Controls
3	Baez et al., 2016 [36]	MRI, atrophy	FTD vs Controls
4	Boccardi et al., 2005 [37]	MRI, atrophy	FTD vs Controls

5	Dermody et al., 2016 [38]	MRI, atrophy	FTD vs Controls
6	Flanagan et al., 2016 [39]	MRI, atrophy	FTD vs Controls
7	Grossman et al., 2004 [30]	MRI, atrophy	FTD vs Controls
8	Irish et al., 2013 [32]	MRI, atrophy	FTD vs Controls
9	Irish et al., 2014 [40]	MRI, atrophy	FTD vs Controls
10	Irish et al., 2016 [41]	MRI, atrophy	FTD vs Controls
11	Kanda et al., 2008 [42]	MRI, atrophy	FTD vs Controls
12	Kipps et al., 2009 [43]	MRI, atrophy	FTD vs Controls
13	Luis et al., 2016 [44]	MRI, atrophy	FTD vs Controls
14	Mandelli et al., 2016 [45]	MRI, atrophy	FTD vs Controls
15	Massimo et al., 2013 [46]	MRI, atrophy	FTD vs Controls
16	Ossenkoppele et al., 2015 [47]	MRI, atrophy	FTD vs Controls
17	Pardini et al., 2009 [48]	MRI, atrophy	FTD vs Controls
18	Tu et al., 2005 [49]	MRI, atrophy	FTD vs Controls
19	Whitwell et al., 2011 [50]	MRI, atrophy	FTD vs Controls
20	Wong et al., 2016 [51]	MRI, atrophy	FTD vs Controls
21	Zamboni et al., 2008 [52]	MRI, atrophy	FTD vs Controls

**Corticobasal Syndrome (CS)**			
1	Boxer et al., 2006 [53]	MRI, atrophy	CS vs Controls
2	Garraux et al, 2000 [54]	MRI, atrophy	CS vs Controls
3	Gross et al., 2010 [55]	MRI, atrophy	CS vs Controls
4	Grossman et al., 2004 [30]	MRI, atrophy	CS vs Controls
5	Huey et al., 2009 [56]	MRI, atrophy	CS vs Controls
6	Hosaka et al., 2002 [57]	MRI, atrophy	CS vs Controls
7	Huh et al., 2005 [58]	MRI, atrophy	CS vs Controls
8	Lee et al., 2011 [59]	MRI, atrophy	CS vs Controls
9	McMillan et al., 2016 [60]	MRI, atrophy	CS vs Controls
10	Morgan et al., 2011 [61]	MRI, atrophy	CS vs Controls
11	Pardini et al., 2009 [48]	MRI, atrophy	CS vs Controls
12	Wolpe et al., 2014 [62]	MRI, atrophy	CS vs Controls
**Progressive Non-Fluent Aphasia (PNFA)**			
1	Gorno-Tempini et al, 2004 [63]	MRI, atrophy	PNFA vs Controls
2	Gorno-Tempini et al, 2006 [64]	MRI, atrophy	PNFA vs Controls
3	Grossman et al., 2004 [30]	MRI, atrophy	PNFA vs Controls

4	Hu et al., 2010 [65]	MRI, atrophy	PNFA vs Controls
5	Nestor et al, 2003	MRI, atrophy	PNFA vs Controls
6	Pereira et al, 2009	MRI, atrophy	PNFA vs Controls
7	Wilson et al, 2010	MRI, atrophy	PNFA vs Controls
8	Zahn et al, 2005	MRI, atrophy	PNFA vs Controls

Supplementary Table 6 Search strategy for simultaneous EEG-fMRI studies

Database	PubMed
Data of search	Initial search November 2020, updated January 2021
Search strategy	Search item:<br>((imaging[Title/Abstract]) OR (magnetic resonance imaging[Title/Abstract])) OR (fMRI[Title/Abstract])) AND ((humans[Filter]) AND (english[Filter])) AND ((Simultaneous[Title/Abstract]) AND ((epilepsy[Title/Abstract]) OR (spike[Title/Abstract]) OR (seizure[Title/Abstract])) AND (EEG[Title/Abstract])<br>Publication data :From 1976.1.1<br>Species:Humans<br>Language:English
Items found	251

Supplementary Table 7. Detailed information for included simultaneous EEG-fMRI studies.

**Reference**	**Sample size**	**Event type**	**Epilepsy subtype**	**P-value**
**Generalized epilepsy**				
Hamandi et al., 2006 [66]	30	GSW	JAE, JME, CAE, GTCS	$P_{uncorr} < 0.001$
Moeller et al., 2008 [67]	10	GSW	CAE	$P_{uncorr} < 0.05$
Carney et al., 2010 [68]	11	GSW	CAE	$P_{uncorr} < 0.001$
Liao et al., 2013 [70]	15	GSW	CAE	$P_{corr} < 0.05$

Moeller et al., 2014 [71]	11	GSW	MAE	$P_{uncorr} < 0.005$
Benuzzi et al., 2015 [72]	21	GSW	IGE	$P_{uncorr} < 0.005$
**Focal epilepsy**				
Laufs et al., 2007 [73]	9	IED	TLE	$P_{corr} < 0.05$
Laufs et al., 2007 [73]	10	IED	Extra-TLE	$P_{uncorr} < 0.001$
Moeller et al., 2013 [74]	10	IED	ABPE	$P_{uncorr} < 0.001$
Wiest ET AL., 2013 [75]	10	IED	TLE	$P_{corr} < 0.05$
Coan et al., 2014 [76]	12	IED	TLE	$P_{uncorr} < 0.005$
Coan et al., 2014 [76]	13	IED	TLE-HS	$P_{uncorr} < 0.005$

ABPE = Atypical benign partial epilepsy; CAE = Childhood absence epilepsy; FOCA = Four-dimensional consistency of local neural activities; GMV = Gray matter volume; GSW = Generalized spike wave; GTCS = Generalized tonic-clonic seizures; IED = Interictal epileptiform discharges; IGE = Idiopathic generalized epilepsy; HS = Hippocampal sclerosis; JAE = Juvenile absence epilepsy; JME = Juvenile myoclonic epilepsy; LGS = Lennox-Gastaut syndrome; MAE = Myoclonic astatic epilepsy.

Supplementary Table 8. Coordinates of SCAN nodes derived from Gordon et al. [77].

Regions	X	Y	Z
**Inter-effectors (M1)**			
Left superior node	-19	-34	59
Right superior node	20	-31	58
Left middle node	-38	-18	44
Right middle node	40	-15	43
Left inferior node	-54	-3	14
Right inferior node	56	-1	16
**Midline (cortex)**			
Left supplementary motor area	-5	-7	52
Right supplementary motor area	5	-5	49
Left dorsal anterior cingulate cortex	-7	1	36
Right dorsal anterior cingulate cortex	6	3	36

**Subcortical structures**			
Left putamen	-28	-5	-1
Rigth putamen	28	-9	3
Left thalamus	-10	-21	2
Right thalamus	12	-20	3
**Cerebellum**			
Left dorsal cerebellum	-9	-65	-18
Right dorsal cerebellum	11	-61	-16
Left ventral cerebellum	-23	-53	-54
Right ventral cerebellum	24	-59	-54

Supplementary Table 9. Coordinates of effector nodes derived from *Yeo et al*.[78]

	Left hemisphere	Right hemisphere
**Motor nodes**		
Foot	-6, -26, 76	6, -26, 76
Hand	-41, -20, 62	41, -20, 62
Tongue	-55, -4, 26	55, -4, 26

Supplementary Table 10. Demographics of 21 patients with IGE treated with CM DBS.

**Subject**	**Sex**	**Age group at surgery**	**Seizure frequency (number/ month)***	**Seizure types**	**Follow-up (months)**	**DBS active contacts**		**DBS amplitude**		**DBS frequency (Hz)**	**DBS pulse width (µs)**	**Seizure reduction after DBS (%)**
						Left	*Right*	*Left*	*Right*	Bilateral	Bilateral	
1	Female	30-40	6	Abs, *GTCS*	11	C+1-	C+1-	2.5 mA	2.5 mA	145	90	92
2	Female	30-40	3	*GTCS*	10	C+2-5-	C+1-4-	3 mA	3 mA	145	90	0
3	Male	20-30	2	*GTCS*	9	C+1-2-3-	C+1-2-3-	3 mA	3 mA	145	90	100
4	Male	>40	6	Abs, Myo, *GTCS*	17	C+1-2-3-	C+1-2-3-	3.5 mA	3.5 mA	145	90	67
5	Non-binary**	30-40	3.5	*GTCS*	21	C+0-	C+1-	4 mA	4 mA	60	90	71
6	Female	30-40	1.5	*GTCS*	13	C+0-	C+0-	4.5	4.5	60	90	100

								mA	mA			
7	Female	20-30	2.5	Abs, Myo, *GTCS*	23	C+2-	C+2-	4 mA	4 mA	7	90	60
8	Female	>40	1	*GTCS*	7	C+1-2-	C+1-2-	2 mA	2 mA	60	90	-200
9	Male	>40	20	Abs, *GTCS*	216	C+0-1-2-	C+0-1-2-	5 V	4.2 V	60	90	100
10	Male	>40	3	*GTCS*	154	0-1-2-3+	0-1-2-3+	3 V	3 V	130	90	90
11	Female	20-30	25	Abs, *GTCS*	135	1-2+	1+2-	4 mA	1.5 mA	60	90	95
12	Female	20-30	10	Abs, *GTCS*	96	1-2+	1-2+	4 mA	4 mA	130	90	70
13	Female	>40	360	*Abs, Myo*	12	1-3+	1-3+	3.3 V	3.3 V	130	200	75
14	Female	>40	26	*Myo, GTCS*	26	C+1-	C+1-	2 V	3.3 V	60	90	92
15	Female	20-30	413	*Abs, Myo, GTCS*	11	1-3+	1-3+	1 V	3.3 V	130	300	64
16	Female	20-30	100	*Abs*	36	0-3+	1-3+	4 V	4 V	130	300	94
17	Female	20-30	400	*Abs*	60	0-3+	0-3+	4.5 V	4.5 V	130	300	93
18	Female	20-30	200	*Abs*	144	0-3+	0-3+	3.5 V	3.5 V	130	300	98
19	Female	20-30	7	*Abs, Myo, GTCS*	36	0-3+	0-3+	3 V	3 V	130	300	75
20	Female	>40	0.3	Abs, Myo, *GTCS*	18	C+2-	C+1-2-	1.5 mA	1 mA	145	90	100
21	Male	>40	3	Myo, *GTCS*	24	C+1-2-	C+9-	4 mA	3.5 mA	145	90	66

Note that 14 of 21 patients were previously published in other papers and collated here, but 7 patients are new previously unpublished cases. While these 14 published patients were described before, all analyses and results are unique to the present paper. *Seizure frequency numbers before surgery refer to the seizure type written in *italic* in the column 'seizure types'. Most patients were able to count generalized tonic clonic seizures (GTCS), but did not keep a seizure diary for absence or myoclonic seizures. ** Patient preferred to report gender instead of sex. *Abbreviations: Abs, absences; Myo, myoclonic; GTCS, generalized tonic clonic seizures.*

Supplementary References:

1. Tahmasian, M., *et al.* Practical recommendations to conduct a neuroimaging meta-analysis for neuropsychiatric disorders. *Human brain mapping* **40**, 5142-5154 (2019).
2. Yang, H., *et al.* Amplitude of low frequency fluctuation within visual areas revealed by resting-state functional MRI. *NeuroImage* **36**, 144-152 (2007).
3. Zang, Y., Jiang, T., Lu, Y., He, Y. & Tian, L. Regional homogeneity approach to fMRI data analysis. *NeuroImage* **22**, 394-400 (2004).
4. Zhang, Z., *et al.* Altered functional-structural coupling of large-scale brain networks in idiopathic generalized epilepsy. *Brain : a journal of neurology* **134**, 2912-2928 (2011).
5. Ji, G.J., *et al.* Identifying Corticothalamic Network Epicenters in Patients with Idiopathic Generalized Epilepsy. *Am J Neuroradiol* **36**, 1494-1500 (2015).
6. Ji, G.J., *et al.* Generalized tonic-clonic seizures: aberrant interhemispheric functional and anatomical connectivity. *Radiology* **271**, 839-847 (2014).
7. Chan, C.H., *et al.* Thalamic atrophy in childhood absence epilepsy. *Epilepsia* **47**, 399-405 (2006).
8. Kim, J.H., *et al.* Regional grey matter abnormalities in juvenile myoclonic epilepsy: a voxel-based morphometry study. *NeuroImage* **37**, 1132-1137 (2007).
9. de Araujo Filho, G.M., *et al.* Personality traits related to juvenile myoclonic epilepsy: MRI reveals prefrontal abnormalities through a voxel-based morphometry study. *Epilepsy Behav* **15**, 202-207 (2009).
10. Huang, W., *et al.* Gray-matter volume reduction in the thalamus and frontal lobe in epileptic patients with generalized tonic-clonic seizures. *J Neuroradiol* **38**, 298-303 (2011).
11. Liu, M., Concha, L., Beaulieu, C. & Gross, D.W. Distinct white matter abnormalities in different idiopathic generalized epilepsy syndromes. *Epilepsia* **52**, 2267-2275 (2011).
12. O'Muircheartaigh, J., *et al.* Focal structural changes and cognitive dysfunction in juvenile myoclonic epilepsy. *Neurology* **76**, 34-40 (2011).
13. Kim, J.H., Kim, J.B., Seo, W.K., Suh, S.I. & Koh, S.B. Volumetric and shape analysis of thalamus in idiopathic generalized epilepsy. *Journal of neurology* **260**, 1846-1854 (2013).
14. Kim, J.B., *et al.* Altered thalamocortical functional connectivity in idiopathic generalized epilepsy. *Epilepsia* **55**, 592-600 (2014).
15. Lin, K., *et al.* Voxel-based morphometry evaluation of patients with photosensitive juvenile myoclonic epilepsy. *Epilepsy research* **86**, 138-145 (2009).
16. Wang, J., Li, Y., Wang, Y. & Huang, W. Multimodal Data and Machine Learning for Detecting Specific Biomarkers in Pediatric Epilepsy Patients With Generalized Tonic-Clonic Seizures. *Frontiers in neurology* **9**, 1038 (2018).
17. Zeng, L.L., *et al.* Gray Matter Loss and Related Functional Connectivity Alterations in A Chinese Family With Benign Adult Familial Myoclonic Epilepsy. *Medicine* **94**, e1767 (2015).
18. Zhong, C., *et al.* Altered Structural and Functional Connectivity of Juvenile Myoclonic Epilepsy: An fMRI Study. *Neural plasticity* **2018**, 7392187 (2018).
19. Zhong, Y., *et al.* Altered regional synchronization in epileptic patients with generalized tonic-clonic seizures. *Epilepsy research* **97**, 83-91 (2011).
20. Jiang, S., *et al.* Altered Local Spontaneous Brain Activity in Juvenile Myoclonic Epilepsy: A Preliminary Resting-State fMRI Study. *Neural plasticity* **2016**, 3547203 (2016).
21. Zhu, L., *et al.* Aberrant long-range functional connectivity density in generalized tonic-clonic

seizures. *Medicine* **95**, e3893 (2016).
22. Ma, S., *et al.* Altered Local Spatiotemporal Consistency of Resting-State BOLD Signals in Patients with Generalized Tonic-Clonic Seizures. *Frontiers in computational neuroscience* **11**, 90 (2017).
23. Jia, X., *et al.* Disrupted Coupling Between the Spontaneous Fluctuation and Functional Connectivity in Idiopathic Generalized Epilepsy. *Frontiers in neurology* **9**, 838 (2018).
24. Liu, G., *et al.* Abnormalities of diffusional kurtosis imaging and regional homogeneity in idiopathic generalized epilepsy with generalized tonic-clonic seizures. *Exp Ther Med* **17**, 603-612 (2019).
25. Yan, Y., Xie, G., Zhou, H., Liu, H. & Wan, M. Altered spontaneous brain activity in patients with childhood absence epilepsy: associations with treatment effects. *Neuroreport* **31**, 613-618 (2020).
26. Baron, J.C., *et al.* In vivo mapping of gray matter loss with voxel-based morphometry in mild Alzheimer's disease. *NeuroImage* **14**, 298-309 (2001).
27. Boxer, A.L., *et al.* Focal right inferotemporal atrophy in AD with disproportionate visual constructive impairment. *Neurology* **61**, 1485-1491 (2003).
28. Boxer, A.L., *et al.* Cinguloparietal atrophy distinguishes Alzheimer disease from semantic dementia. *Archives of neurology* **60**, 949-956 (2003).
29. Bozzali, M., *et al.* The contribution of voxel-based morphometry in staging patients with mild cognitive impairment. *Neurology* **67**, 453-460 (2006).
30. Grossman, M., *et al.* What's in a name: voxel-based morphometric analyses of MRI and naming difficulty in Alzheimer's disease, frontotemporal dementia and corticobasal degeneration. *Brain : a journal of neurology* **127**, 628-649 (2004).
31. Ohnishi, T., Matsuda, H., Tabira, T., Asada, T. & Uno, M. Changes in brain morphology in Alzheimer disease and normal aging: is Alzheimer disease an exaggerated aging process? *AJNR. American journal of neuroradiology* **22**, 1680-1685 (2001).
32. Irish, M., Hodges, J.R. & Piguet, O. Episodic future thinking is impaired in the behavioural variant of frontotemporal dementia. *Cortex; a journal devoted to the study of the nervous system and behavior* **49**, 2377-2388 (2013).
33. Zahn, R., *et al.* Mapping of temporal and parietal cortex in progressive nonfluent aphasia and Alzheimer's disease using chemical shift imaging, voxel-based morphometry and positron emission tomography. *Psychiatry research* **140**, 115-131 (2005).
34. Amanzio, M., *et al.* Neural correlates of reduced awareness in instrumental activities of daily living in frontotemporal dementia. *Exp Gerontol* **83**, 158-164 (2016).
35. Baez, S., *et al.* Integration of Intention and Outcome for Moral Judgment in Frontotemporal Dementia: Brain Structural Signatures. *Neurodegener Dis* **16**, 206-217 (2016).
36. Baez, S., *et al.* Orbitofrontal and limbic signatures of empathic concern and intentional harm in the behavioral variant frontotemporal dementia. *Cortex; a journal devoted to the study of the nervous system and behavior* **75**, 20-32 (2016).
37. Boccardi, M., *et al.* Frontotemporal dementia as a neural system disease. *Neurobiology of aging* **26**, 37-44 (2005).
38. Dermody, N., Hornberger, M., Piguet, O., Hodges, J.R. & Irish, M. Prospective Memory Impairments in Alzheimer's Disease and Behavioral Variant Frontotemporal Dementia: Clinical and Neural Correlates. *J Alzheimers Dis* **50**, 425-441 (2016).

39. Flanagan, E.C., *et al.* False Recognition in Behavioral Variant Frontotemporal Dementia and Alzheimer's Disease-Disinhibition or Amnesia? *Frontiers in aging neuroscience* **8**, 177 (2016).
40. Irish, M., *et al.* Grey and white matter correlates of recent and remote autobiographical memory retrieval--insights from the dementias. *PloS one* **9**, e113081 (2014).
41. Irish, M., *et al.* Neural Substrates of Semantic Prospection - Evidence from the Dementias. *Front Behav Neurosci* **10**, 96 (2016).
42. Kanda, T., *et al.* Comparison of grey matter and metabolic reductions in frontotemporal dementia using FDG-PET and voxel-based morphometric MR studies. *Eur J Nucl Med Mol Imaging* **35**, 2227-2234 (2008).
43. Kipps, C.M., Nestor, P.J., Acosta-Cabronero, J., Arnold, R. & Hodges, J.R. Understanding social dysfunction in the behavioural variant of frontotemporal dementia: the role of emotion and sarcasm processing. *Brain : a journal of neurology* **132**, 592-603 (2009).
44. Luis, E., *et al.* Neuroimaging Correlates of Frontotemporal Dementia Associated with SQSTM1 Mutations. *J Alzheimers Dis* **53**, 303-313 (2016).
45. Mandelli, M.L., *et al.* Two insular regions are differentially involved in behavioral variant FTD and nonfluent/agrammatic variant PPA. *Cortex; a journal devoted to the study of the nervous system and behavior* **74**, 149-157 (2016).
46. Massimo, L., *et al.* Self-appraisal in behavioural variant frontotemporal degeneration. *Journal of neurology, neurosurgery, and psychiatry* **84**, 148-153 (2013).
47. Ossenkoppele, R., *et al.* The behavioural/dysexecutive variant of Alzheimer's disease: clinical, neuroimaging and pathological features. *Brain : a journal of neurology* **138**, 2732-2749 (2015).
48. Pardini, M., Huey, E.D., Cavanagh, A.L. & Grafman, J. Olfactory function in corticobasal syndrome and frontotemporal dementia. *Archives of neurology* **66**, 92-96 (2009).
49. Tu, S., *et al.* Lost in spatial translation - A novel tool to objectively assess spatial disorientation in Alzheimer's disease and frontotemporal dementia. *Cortex; a journal devoted to the study of the nervous system and behavior* **67**, 83-94 (2015).
50. Whitwell, J.L., *et al.* Temporoparietal atrophy: a marker of AD pathology independent of clinical diagnosis. *Neurobiology of aging* **32**, 1531-1541 (2011).
51. Wong, S., *et al.* Comparison of Prefrontal Atrophy and Episodic Memory Performance in Dysexecutive Alzheimer's Disease and Behavioral-Variant Frontotemporal Dementia. *J Alzheimers Dis* **51**, 889-903 (2016).
52. Zamboni, G., Huey, E.D., Krueger, F., Nichelli, P.F. & Grafman, J. Apathy and disinhibition in frontotemporal dementia: Insights into their neural correlates. *Neurology* **71**, 736-742 (2008).
53. Boxer, A.L., *et al.* Patterns of brain atrophy that differentiate corticobasal degeneration syndrome from progressive supranuclear palsy. *Archives of neurology* **63**, 81-86 (2006).
54. Garraux, G., *et al.* Voxel-based distribution of metabolic impairment in corticobasal degeneration. *Movement disorders : official journal of the Movement Disorder Society* **15**, 894-904 (2000).
55. Gross, R.G., *et al.* Impaired information integration contributes to communication difficulty in corticobasal syndrome. *Cogn Behav Neurol* **23**, 1-7 (2010).
56. Huey, E.D., *et al.* Executive dysfunction in frontotemporal dementia and corticobasal syndrome. *Neurology* **72**, 453-459 (2009).
57. Hosaka, K., *et al.* Voxel-based comparison of regional cerebral glucose metabolism between PSP and corticobasal degeneration. *Journal of the neurological sciences* **199**, 67-71 (2002).

58. Juh, R., *et al.* Cerebral glucose metabolism in corticobasal degeneration comparison with progressive supranuclear palsy using statistical mapping analysis. *Neuroscience letters* **383**, 22-27 (2005).
59. Lee, S.E., *et al.* Clinicopathological correlations in corticobasal degeneration. *Annals of neurology* **70**, 327-340 (2011).
60. McMillan, C.T., *et al.* Multimodal imaging evidence of pathology-mediated disease distribution in corticobasal syndrome. *Neurology* **87**, 1227-1234 (2016).
61. Morgan, B., *et al.* Some is not enough: quantifier comprehension in corticobasal syndrome and behavioral variant frontotemporal dementia. *Neuropsychologia* **49**, 3532-3541 (2011).
62. Wolpe, N., *et al.* The medial frontal-prefrontal network for altered awareness and control of action in corticobasal syndrome. *Brain : a journal of neurology* **137**, 208-220 (2014).
63. Gorno-Tempini, M.L., *et al.* Cognition and anatomy in three variants of primary progressive aphasia. *Annals of neurology* **55**, 335-346 (2004).
64. Gorno-Tempini, M.L., *et al.* Anatomical correlates of early mutism in progressive nonfluent aphasia. *Neurology* **67**, 1849-1851 (2006).
65. Hu, W.T., *et al.* Multimodal predictors for Alzheimer disease in nonfluent primary progressive aphasia. *Neurology* **75**, 595-602 (2010).
66. Hamandi, K., *et al.* EEG-fMRI of idiopathic and secondarily generalized epilepsies. *NeuroImage* **31**, 1700-1710 (2006).
67. Moeller, F., *et al.* Simultaneous EEG-fMRI in drug-naive children with newly diagnosed absence epilepsy. *Epilepsia* **49**, 1510-1519 (2008).
68. Carney, P.W., *et al.* The core network in absence epilepsy. Differences in cortical and thalamic BOLD response. *Neurology* **75**, 904-911 (2010).
69. Siniatchkin, M., Coropceanu, D., Moeller, F., Boor, R. & Stephani, U. EEG-fMRI reveals activation of brainstem and thalamus in patients with Lennox-Gastaut syndrome. *Epilepsia* **52**, 766-774 (2011).
70. Liao, W., *et al.* Dynamical intrinsic functional architecture of the brain during absence seizures. *Brain structure & function* **219**, 2001-2015 (2013).
71. Moeller, F., *et al.* EEG-fMRI in myoclonic astatic epilepsy (Doose syndrome). *Neurology* **82**, 1508-1513 (2014).
72. Benuzzi, F., *et al.* An EEG-fMRI Study on the Termination of Generalized Spike-And-Wave Discharges in Absence Epilepsy. *PloS one* **10**, e0130943 (2015).
73. Laufs, H., *et al.* Temporal lobe interictal epileptic discharges affect cerebral activity in "default mode" brain regions. *Human brain mapping* **28**, 1023-1032 (2007).
74. Moeller, F., *et al.* EEG-fMRI in atypical benign partial epilepsy. *Epilepsia* **54**, e103-108 (2013).
75. Wiest, R., *et al.* Widespread grey matter changes and hemodynamic correlates to interictal epileptiform discharges in pharmacoresistant mesial temporal epilepsy. *Journal of neurology* **260**, 1601-1610 (2013).
76. Coan, A.C., *et al.* Distinct functional and structural MRI abnormalities in mesial temporal lobe epilepsy with and without hippocampal sclerosis. *Epilepsia* **55**, 1187-1196 (2014).
77. Gordon, E.M., *et al.* A somato-cognitive action network alternates with effector regions in motor cortex. *Nature* **617**, 351-359 (2023).
78. Yeo, B.T., *et al.* The organization of the human cerebral cortex estimated by intrinsic functional connectivity. *Journal of neurophysiology* **106**, 1125-1165 (2011).
