# Supplementary material for: A generalized epilepsy network derived from brain abnormalities and deep brain stimulation
Source: Nat Commun. 2025 Mar 24;16:2783. doi: 10.1038/s41467-025-57392-7 (PMC11933423; doi:10.1038/s41467-025-57392-7)
Supplement: Supplementary file 2 — Reporting Summary [file 41467_2025_57392_MOESM2_ESM.pdf]

nature portfolio

Corresponding author(s): Kai Wang, Frederic L.W.V.J Schaper

Last updated by author(s): Jan 1, 2025

Reporting Summary

Nature Portfolio wishes to improve the reproducibility of the work that we publish. This form provides structure for consistency and transparency in reporting. For further information on Nature Portfolio policies, see our Editorial Policies and the Editorial Policy Checklist.

Statistics

For all statistical analyses, confirm that the following items are present in the figure legend, table legend, main text, or Methods section.

n/a	Confirmed	
☐	☒	The exact sample size (n) for each experimental group/condition, given as a discrete number and unit of measurement
☐	☒	A statement on whether measurements were taken from distinct samples or whether the same sample was measured repeatedly
☐	☒	The statistical test(s) used AND whether they are one- or two-sided<br>*Only common tests should be described solely by name; describe more complex techniques in the Methods section.*
☐	☒	A description of all covariates tested
☐	☒	A description of any assumptions or corrections, such as tests of normality and adjustment for multiple comparisons
☐	☒	A full description of the statistical parameters including central tendency (e.g. means) or other basic estimates (e.g. regression coefficient) AND variation (e.g. standard deviation) or associated estimates of uncertainty (e.g. confidence intervals)
☐	☒	For null hypothesis testing, the test statistic (e.g. F, t, r) with confidence intervals, effect sizes, degrees of freedom and P value noted<br>*Give P values as exact values whenever suitable.*
☒	☐	For Bayesian analysis, information on the choice of priors and Markov chain Monte Carlo settings
☒	☐	For hierarchical and complex designs, identification of the appropriate level for tests and full reporting of outcomes
☐	☒	Estimates of effect sizes (e.g. Cohen's d, Pearson's r), indicating how they were calculated

Our web collection on statistics for biologists contains articles on many of the points above.

Software and code

Policy information about availability of computer code

Data collection	All the functional imaging data were collected on 3T MRI scanners and preprocessed consistent with prior published and validated methods.
Data analysis	Code to conduct preprocessing and connectivity analyses is available as part of the open-access Lead-DBS software package (https://lead-dbs.org) and WhiteMatterSF software (https://github.com/jigongjun/Neuroimaging-and-Neuromodulation).

For manuscripts utilizing custom algorithms or software that are central to the research but not yet described in published literature, software must be made available to editors and reviewers. We strongly encourage code deposition in a community repository (e.g. GitHub). See the Nature Portfolio guidelines for submitting code & software for further information.

Data

Policy information about availability of data

All manuscripts must include a data availability statement. This statement should provide the following information, where applicable:

- Accession codes, unique identifiers, or web links for publicly available datasets
- A description of any restrictions on data availability
- For clinical datasets or third party data, please ensure that the statement adheres to our policy

All coordinates of grey matter atrophy and fMRI hyperactivity used in this study are available in the published studies listed in supplementary materials. A version of the GSP connectome along with preprocessing details is publicly available (doi:10.7910).

Research involving human participants, their data, or biological material

Policy information about studies with human participants or human data. See also policy information about sex, gender (identity/presentation), and sexual orientation and race, ethnicity and racism.

Reporting on sex and gender	MRI data of 652 participants (316 males and 336 females) were collected at Anhui Medical University (Hefei, China).MRI data of 52 epilepsy patients (31 males and 21 females; age, 30.0±10.74 years) were obtained at the First Affiliated Hospital of Anhui Medical University (Hefei, China). The epilepsy data of 120 patients (81 males and 39 females; age, 25.5±8.68 years) were acquired on a clinical 3-T MR scanner at Jinling Hospital (NanJing, China).
Reporting on race, ethnicity, or other socially relevant groupings	We have replicated our results using normative connectomes derived from different ethnicities (Western and Asian adult populations) and have reported the findings in the result section.
Population characteristics	We performed a systematic search for MRI studies comparing patients with idiopathic generalized epilepsy to healthy controls. Coordinates of structural abnormalities associated with idiopathic generalized epilepsy derived from these studies were used to identify a generalized epilepsy network. The population characteristics are described in each original study listed in our supplemental materials.
Recruitment	Patients or healthy participants were recruited without predefined bias.
Ethics oversight	The study was approved by the IRB at Brigham and Women's Hospital (Protocol no. 2020P002987)

Note that full information on the approval of the study protocol must also be provided in the manuscript.

Field-specific reporting

Please select the one below that is the best fit for your research. If you are not sure, read the appropriate sections before making your selection.

☒ Life sciences ☐ Behavioural & social sciences ☐ Ecological, evolutionary & environmental sciences

For a reference copy of the document with all sections, see nature.com/documents/nr-reporting-summary-flat.pdf

Life sciences study design

All studies must disclose on these points even when the disclosure is negative.

Sample size	Because there is no standard method for estimating sample size for this type of study, we aimed to identify as many samples as possible across multiple centers to maximize power. The normative connectome data were were from three groups of healthy controls (n1=652, n2=1000, n3=1000), and an idiopathic generalized epilepsy group (n=172). We performed a systematic search to include all published structural and functional MRI studies of comparing patients with idiopathic generalized epilepsy to healthy controls. As a result, 21 studies were finally included in this study.
Data exclusions	All studies that fit our in- and exclusion criteria listed in the methods were included. Functional imaging data of all subjects with complete neuroimaging were included.
Replication	As outlined in the manuscript, we the results were independent of variations and w e provided multimodal support for the results using both neuroimaging and DBS data. Results were replicated using multiple different normative and disease specific connectomes.
Randomization	Rather than prospective randomization, this retrospective study capitalized on incidental variability of coordinates from different published studies.
Blinding	Blinding was not relevant because this was a secondary analysis of existing datasets. We mitigated the risk of observer bias by testing our previously-published hypothesis (Fox et al, PNAS 2014) in multiple independent datasets.

Reporting for specific materials, systems and methods

We require information from authors about some types of materials, experimental systems and methods used in many studies. Here, indicate whether each material, system or method listed is relevant to your study. If you are not sure if a list item applies to your research, read the appropriate section before selecting a response.

Materials & experimental systems

n/a	Involved in the study
☒	☐ Antibodies
☒	☐ Eukaryotic cell lines
☒	☐ Palaeontology and archaeology
☒	☐ Animals and other organisms
☒	☐ Clinical data
☒	☐ Dual use research of concern
☒	☐ Plants

Methods

n/a	Involved in the study
☒	☐ ChIP-seq
☒	☐ Flow cytometry
☐	☒ MRI-based neuroimaging

Plants

Seed stocks

Not available.

Novel plant genotypes

Not available.

Authentication

Not available.

Magnetic resonance imaging

Experimental design

Design type

Resting-state functional MRI data

Design specifications

Resting -state functional MRI data from three healthy groups and one epilepsy group were used to estimate functional connectivity of each coordinate published in the literature.

Behavioral performance measures

None. Resting state functional MRI was acquired.

Acquisition

Imaging type(s)

resting-state functional and structural MRI

Field strength

3 Tesla

Sequence & imaging parameters

Images of healthy participants at Anhui Medical University with 3-T GE Scanner. High resolution T1-weighted images were acquired in the sagittal orientation using a three-dimensional brain-volume sequence (repetition/echo time, 8.16/3.18 ms; flip angle, 12; field of view, 256 mm × 256 mm; 256 × 256 matrix; section thickness, 1 mm; voxel size, 1 mm × 1 mm × 1 mm). Resting-state functional images were acquired using a single shot gradient-recalled echo planar imaging sequence (repetition/echo time, 2400/30 ms; flip angle, 90; field of view, 192 mm × 192 mm; 64 × 64 in-plane matrix; section thickness, 3 mm; voxel size, 3 mm × 3 mm × 3 mm; 46 transverse sections). A total of 217 volumes were acquired (~8.7 mins).MRI data of 52 patients were obtained at the First Affiliated Hospital of Anhui Medical University (Hefei, China) with Siemens 3-T MRI Scanner Prisma. High resolution T1-weighted images were acquired in the sagittal orientation using a magnetization-prepared rapid gradient-echo sequence (repetition/echo time, 2300/2.96 ms; flip angle, 9; field of view, 240 mm × 256 mm; 240 × 256 matrix; section thickness, 1 mm; voxel size, 1 mm × 1 mm × 1 mm). Resting-state functional images were acquired using a single shot gradient-recalled echo planar imaging sequence (repetition/echo time, 3000/30 ms; flip angle, 90; field of view, 220 mm × 220 mm; 64 × 64 in-plane matrix; section thickness, 3.4 mm; voxel size, 3.4 mm × 3.4 mm × 3.4 mm; 48 transverse sections). A total of 217 volumes were acquired (~8.7 mins).Data from Jinling Hospital (NanJing, China) were acquired on a clinical 3-T MR scanner (TIM Trio; Siemens Medical Solutions, Erlangen, Germany) . Functional images were acquired by using a single shot, gradient-recalled echo-planar imaging sequence (repetition time msec/echo time msec, 2000/30; flip angle, 90°, voxel size, 3.75 mm × 3.75 mm × 4.4 mm, 250 volume), aligned along the anterior–posterior commissure line were acquired for each subject, a total of 250 volumes were acquired, The high-spatial-resolution three-dimensional T1-weighted anatomic images were acquired in sagittal orientation by using a magnetization-prepared rapid gradient-echo sequence (repetition/echo time, 2300/2.98; flip angle, 9°; voxel size, 1 mm × 1 mm × 1 mm; sections, 176). See details of the diagnosis and scanning parameters in our previous work

Area of acquisition

Whole brain acquisition.

Diffusion MRI ☐ Used ☒ Not used

Preprocessing

Preprocessing software	SPM12, AFNI and in-house preprocessing scripts (Ji et al., Nat Mental Health, 2023), consistent with the GSP1000 dataset (details in Yeo et al, J Neurophysiol 2011) and the publicly available preprocessed GSP connectome (doi:10.7910)
Normalization	Nonlinear volume-based registration as in Friston et al, 1995
Normalization template	MNI ICBM152
Noise and artifact removal	Low-pass temporal filtering, head-motion regression, global signal regression, and ventricular and white matter signal regression
Volume censoring	Motion regression

Statistical modeling & inference

Model type and settings	Coordinate network mapping with voxel-wise regression model (details described in manuscript).
Effect(s) tested	None. Resting state functional MRI was acquired.

Specify type of analysis: ☒ Whole brain ☐ ROI-based ☐ Both

Statistic type for inference (See Eklund et al. 2016)	voxel wise
Correction	FDR

Models & analysis

n/a	Involved in the study
☐	☒ Functional and/or effective connectivity
☒	☐ Graph analysis
☒	☐ Multivariate modeling or predictive analysis

Functional and/or effective connectivity	Pearson correlations between coordinates and each individual voxel in the whole brain.
